# Characterization of Proteinuria in Dogue de Bordeaux Dogs, a Breed Predisposed to a Familial Glomerulonephropathy: A Retrospective Study

**DOI:** 10.1371/journal.pone.0133311

**Published:** 2015-07-16

**Authors:** Rachel Lavoué, Catherine Trumel, Pascale M. Y. Smets, Jean-Pierre Braun, Luca Aresu, Sylvie Daminet, Didier Concordet, Florence Palanché, Dominique Peeters

**Affiliations:** 1 Internal Medicine Unit, Institut National Polytechnique, Ecole Nationale Vétérinaire de Toulouse (INP-ENVT), Toulouse, France; 2 Université de Toulouse, UPS, INP, ENVT, UMS006, Laboratoire Central de Biologie Médicale, Toulouse, France; 3 INSERM, UMS 006, Laboratoire Central de Biologie Médicale, Toulouse, France; 4 Department of Medicine and Clinical Biology of Small Animals, Faculty of Veterinary Medicine, Ghent University, Merelbeke, Belgium; 5 Department of Comparative Biomedicine and Food Science, University of Padova, Padova, Italy; 6 UMR 1331 Toxalim, INRA, Institut National Polytechnique, Ecole Nationale Vétérinaire de Toulouse (INP-ENVT), Toulouse, France; 7 Department of Companion Animal Clinical Sciences, Faculty of Veterinary Medicine, University of Liège, Liège, Belgium; Fondazione IRCCS Ospedale Maggiore Policlinico & Fondazione D’Amico per la Ricerca sulle Malattie Renali, ITALY

## Abstract

Dogue de Bordeaux dog has been reported to be predisposed to a familial glomerulonephropathy that displays some morphological modifications reported in focal and segmental glomerulosclerosis. Prevalence of quantitatively abnormal renal proteinuria was recently reported to be 33% in this breed. The nature of the proteinuria was assessed by sodium dodecyl sulfate-agarose gel electrophoresis and determinations of urinary markers (urinary retinol-binding protein, urinary N-acetyl-β-glucosaminidase, urinary albumin and urinary immunoglobulin G) on stored specimens. Diagnostic performances of sodium dodecyl sulfate-agarose gel electrophoresis to identify dogs with elevated urinary biomarkers were assessed. Samples from 102 adult Dogue de Bordeaux dogs (47 non-proteinuric [urine protein-to-creatinine ratio≤0.2], 20 borderline-proteinuric [0.2< urine protein-to-creatinine ratio ≤0.5] and 35 proteinuric dogs [urine protein-to-creatinine ratio >0.5]) were used, of which 2 were suffering from familial glomerulonephropathy. The electrophoretic protein patterns, for all but one proteinuric dog, were indicative of a glomerular origin and, in all dogs, the urinary albumin concentration related to creatinine concentration and the urinary immunoglobulin G concentration related to creatinine concentration were above the upper limit of the reference interval established for the breed. Sensitivity and specificity of sodium dodecyl sulfate-agarose gel electrophoresis identifying dogs with elevated urinary albumin concentration were 94% and 92%, respectively, while diagnostic performance of sodium dodecyl sulfate-agarose gel electrophoresis in detecting dogs with elevated urinary immunoglobulin G concentration yielded sensitivity and specificity of 90% and 74%, respectively. These results suggest that all proteinuric and some borderline-proteinuric Dogue de Bordeaux dogs likely have underlying glomerular lesions and that sodium dodecyl sulfate-agarose gel electrophoresis and urinary markers might be useful to screen dogs with borderline-proteinuria. Additional investigations are warranted to assess if these findings are related to the familial glomerulonephropathy.

## Introduction

Dogue de Bordeaux (DDB) dogs can be affected by a familial glomerulonephropathy (GN) that can rapidly lead to end-stage renal failure [1]. A recent observational cross-sectional prospective study revealed that among a cohort of 100 clinically healthy adult DDB dogs, 2 were suffering from proteinuric chronic kidney disease (CKD), while a spot urine sample revealed proteinuria in 33 dogs [2]. As proteinuria is one of the earliest modifications found in some canine familial glomerular diseases [3,4], such proteinuric DDB dogs could be affected by a subclinical form of familial GN. Early lesions in affected animals are characterized by expansion of the matrix, together with focal and segmental distribution, suggesting that primary abnormalities are located in the mesangium. Similar morphological modifications are reported in human focal and segmental glomerulosclerosis (FSGS) [1]. However, FSGS has only been described sporadically in dogs [5].

Human FSGS is a frequent cause of renal disease that is characterized by various morphologic patterns, and a wide range of origins and clinical presentations have been described [6–8]. To date, no spontaneous or experimental animal disease that mimics human FSGS has been reported [9]. In this context, further characterization of proteinuria in clinically healthy DDB dogs is important.

Morphological evaluation is considered the gold standard for characterizing renal lesions and diagnosing GN in dog. However, obtaining renal tissue is invasive and not always recommended, especially in dogs with mildly elevated urine protein-to-creatinine ratio (UPC) [10,11]. In the past decades, promising results have been obtained with urinary proteomic analyses to detect kidney dysfunction in the dog and to characterize renal lesions. The use of such methods to further characterize the origin of proteinuria in DDB dogs is consequently an attractive option.

Both urinary protein electrophoresis and quantitative urinary markers have been used in humans and in dogs to characterize and localize kidney lesions [12–17]. According to several canine studies involving urinary proteins electrophoresis, the presence of albumin bands (Ab) associated with high molecular-weight bands (HMWb) relative to albumin is generally attributed to glomerular lesions, while the presence of low molecular-weight (LMW) urine proteins is supposedly linked to tubular dysfunction [12–14,18–20]. Measurement of some quantitative urinary markers has also been used to localize the origin of proteinuria. These include: urinary retinol-binding protein (uRBP) and urinary enzyme N-acetyl-β-glucosaminidase activity (uNAG) that likely reflect a tubular dysfunction and damage of epithelial tubular cells, respectively [21,22]. On the other hand, elevated concentrations of urinary albumin (uAlb) and immunoglobulin G (uIgG) are mainly indicative of an impaired glomerular barrier [10,23].

The use of urinary protein gel-electrophoresis to predict the localization of the renal lesions by histology has been reported to be highly sensitive (82 to 100%), but poorly specific (40 to 62%) [14,20]. Additionally, several human and veterinary studies have demonstrated good correlations between the concentrations of some urinary markers on the one hand, and the localization and severity of renal histopathological lesions on the other [15,16,24–26]. As urine can be easily obtained, proteinuria characterization seems a promising alternative option for the early detection and localization of underlying renal lesions. While quantitative measurement of urinary markers is not routinely available in veterinary medicine, gel-electrophoresis is inexpensive and readily performed. However, to the best of our knowledge, no direct comparative of urinary protein gel-electrophoresis with quantitative measurement of urinary biomarkers has been carried out in dogs.

Therefore, the aims of the present study were to 1) better characterize the origin of the proteinuria in clinically healthy DDB dogs using electrophoresis, 2) investigate the hypothesis that proteinuria in DDB dogs originates from glomerular alterations quantifying uRBP, uNAG, uAlb and uIgG, 3) identify possible correlations between quantitative and qualitative results, and 4) determine the performance of urine protein electrophoresis to discriminate DDB dogs with abnormal uRBP, uNAG, uAlb and/or uIgG.

## Materials and Methods

### Study design

The present study was performed retrospectively on -80°C stored urine specimens from 100 clinically healthy adult DDB dogs from French and Belgian breeders. These dogs had been initially recruited for hematological and biochemical reference intervals determination study [2,27]. This study was carried out in strict accordance with the recommendations in the Guide for the Care and Use of Laboratory Animals of the National Institutes of Health. The protocol was approved by the Comité d’Ethique Midi Pyrénées pour l’Expérimentation Animale (n° 01). Each dog underwent thorough physical examination, blood sampling, renal ultrasound and cystocentesis. The duration of urine storage prior to the analyses was recorded for each specimen.

Two DDB dogs diagnosed with stage II and stage III proteinuric CKD (International Renal Interest Society (IRIS) staging system) were also included in the study for comparison with results obtained in apparently clinically healthy dogs [28]. When conceivable, renal biopsies were also performed to investigate persistent proteinuria, with or without azotemia, as recommended in the international consensus statement [10]. Briefly, screening tests (systolic blood pressure evaluation, coagulation testing) were systematically performed to exclude contraindications for the procedure, and renal biopsies were obtained percutaneously under ultrasonographic guidance, with a 16G needle. Anesthesia was induced with IV propofol and maintained with inhaled isoflurane. Sections from each specimen were stained with hematoxylin and eosin, periodic acid-Shiff, Masson’s Trichrome and periodic acid methenamine silver for light microscopy examination and glutaraldehyde-fixed tissue were used for transmission electron microscopy.

### Clinically healthy dogs

**Fig 1:**
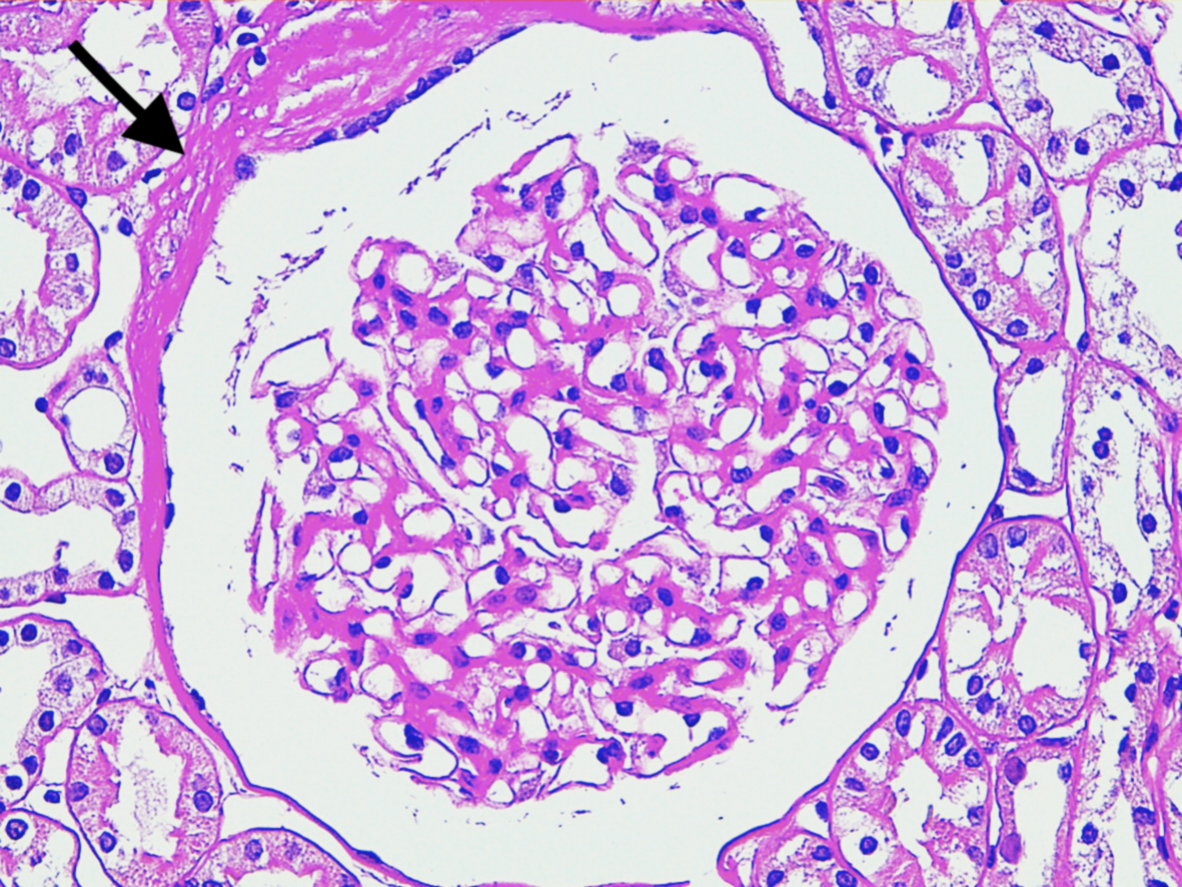
Glomerulus from the non azotemic Dogue de Bordeaux dog. Mild global increase of mesangial matrix, mild increase of Bowman’s space and thickening of Bowman’s capsule (arrow) are visible. Periodic acid—Schiff staining (400x).

**Fig 2:**
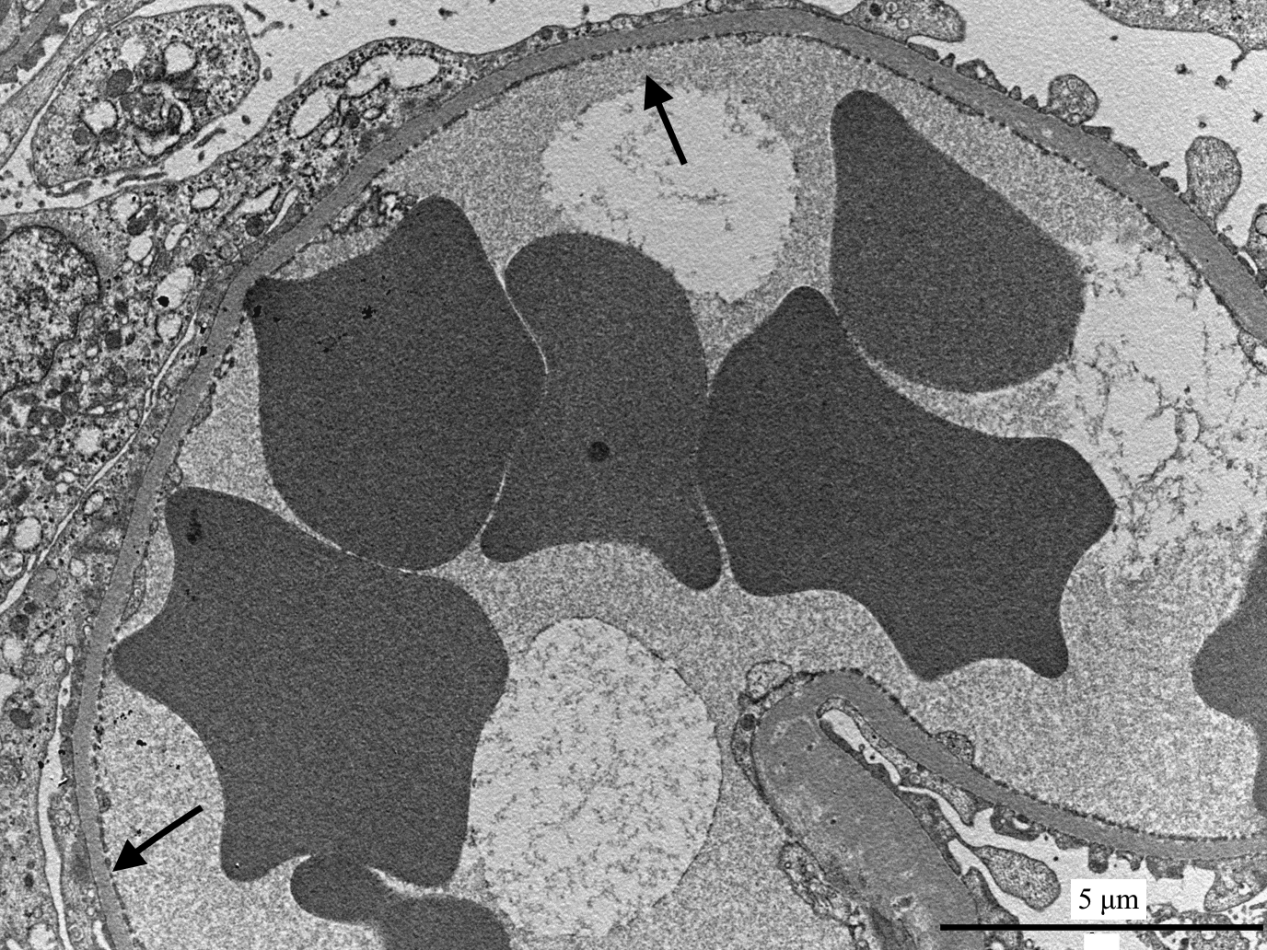
Glomerular capillary from the non azotemic Dogue de Bordeaux dog. Capillary lumen is open, and podocyte foot processes are segmentally effaced (arrows). Transmission electron microscopy.

The initial cohort of clinically healthy dogs consisted of 70 female and 30 male dogs. The hematology and biochemistry results were generally unremarkable, except for a few clinically irrelevant changes when compared to the breed-specific RI [2,27]. Blood-work and renal ultrasound findings were unremarkable in all dogs. However, 47, 20 and 33 dogs had UPC≤0.2 (non-proteinuric), 0.2 < UPC ≤0.5 (borderline proteinuric) and UPC>0.5 (proteinuric), respectively. One dog with persistent UPC >2 underwent renal biopsies [11]. Light microscopy and transmission electron microscopy revealed a global increase of mesangial matrix at the vascular pole; moderate cystic glomerular atrophy and multifocal mild chronic interstitial nephritis associated with tubular degeneration (Figs 1 and 2) and segmental area of podocyte effacement (Fig 2). These results were compatible with an early stage of familial juvenile GN [1].

### Dogs with azotemic chronic kidney disease

**Fig 3:**
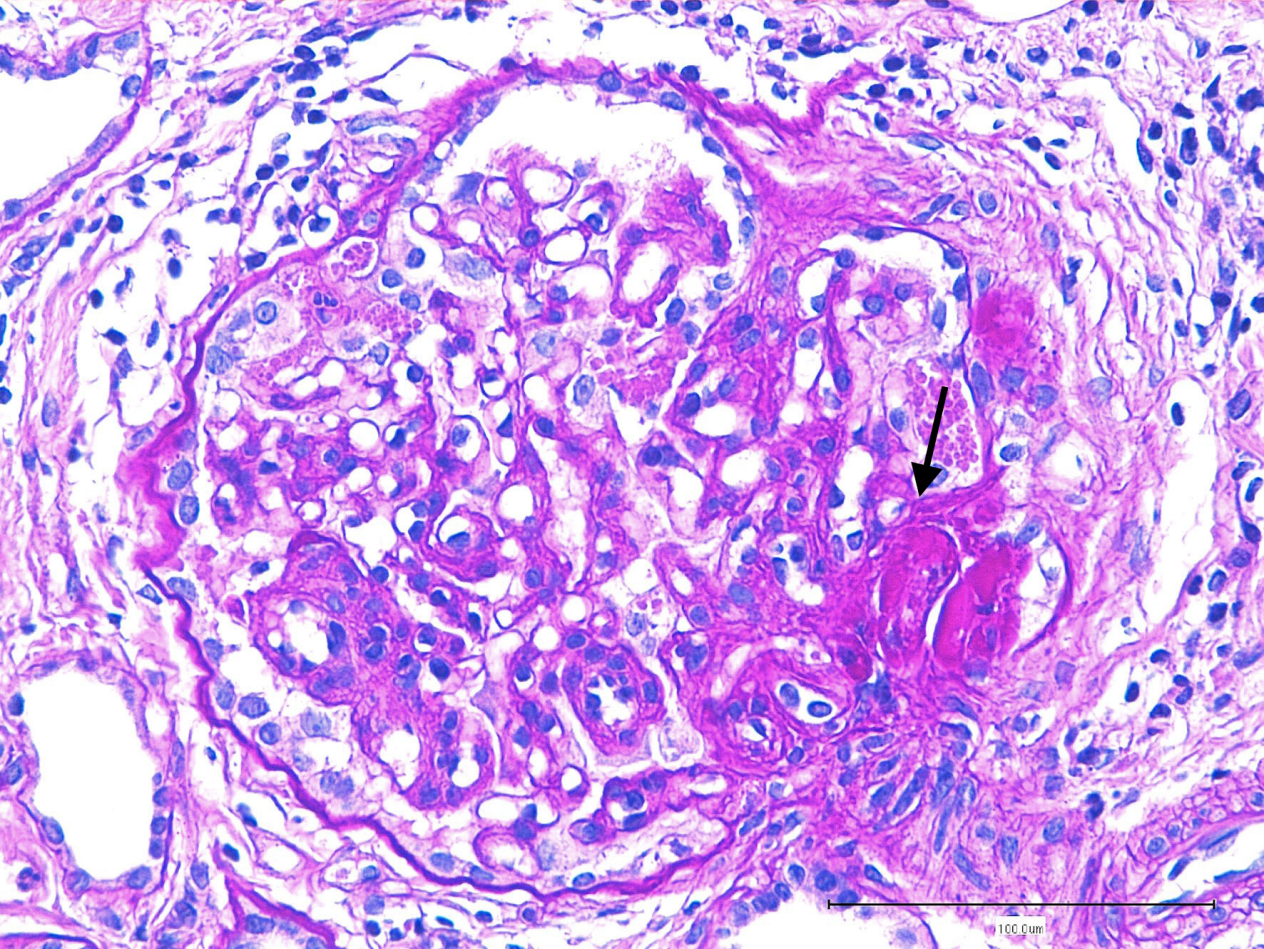
Glomerulus from the azotemic Dogue de Bordeaux dog. Global consolidation of capillary lumens by extracellular matrix, synechiae and deposition of hyaline material (arrow) are visible. Parietal epithelium and podocytes are hypertrophied. Periodic acid—Schiff staining (400x).

The 2 dogs diagnosed with azotemic proteinuric CKD were 3 year-old female dogs. Despite being clinically healthy, both dogs showed renal changes on abdominal ultrasound examination with thickened and hyperechoic cortices and poor delineation of the corticomedullary junction. Both dogs had repeated UPC values > 1, with normal urinary sediment and urine specific gravity <1.020. One dog had a mild normochromic normocytic non regenerative anemia (RBC = 5.01x10^6^/μL, reference interval [5.30–8.48]; Hb = 119 g/L, [128–206]; MCV = 67.1 fL, [61.7–73.2]; MCHC = 353g/L, [347–382]). Both dogs had elevated plasma creatinine (268.6 and 138.7 μmol/L, [74.7–133.1]) and urea concentration (19.3 and 9.0 mmol/L, respectively, [2.2–7.1]) and the anemic dog also had mild hypoalbuminemia (27 g/L [28–39]). The remaining of the biochemical panel was within the previously established breed-specific reference interval [2,27]. Urinary culture, leptospirosis PCR, indirect immunofluorescence serology for Leishmania *infantum*, ELISA serology for Borrelia *burgdorferi*, Ehrlichia *canis* and Anaplasma *phagocytophilum* and ELISA antigen test for Dirofilaria *immitis* (Idexx Test SNAP 4Dx, Idexx Laboratories, France) were negative. Renal biopsies were obtained from the IRIS stage III CKD dog. No glomerulus was available for transmission electron microscopy examination but changes seen under light microscopy were compatible with familial GN. Main lesions were characterized by mesangial proliferation, thickened glomerular basement membrane, hyaline deposition in the mesangium and podocyte hypertrophy with mild multifocal interstitial fibrosis and lymphoplasmacytic nephritis (Fig 3).

### Specimen collection and processing

Urine specimens were obtained from each dog by ultrasound-guided cystocentesis. The urinalysis and urine microscopic examinations were performed immediately after collection. The remaining urine was centrifuged. Supernatant was transferred into plastic cryotubes and immediately stored at -20°C until shipment to the laboratory on dry ice. Upon reception within 24 hours of collection, specimens were stored at -80°C until analyzed. Urinary proteins and creatinine concentrations were respectively measured by pyrogallol red and kinetic Jaffé methods using an automated analyzer (KBio 2 Kitvia, France). For each dog, UPC was determined within 2 weeks of storage. Analytic characteristics of urinary proteins and creatinine assays are reported elsewhere [2].

### Sodium Dodecyl Sulfate-Agarose Gel Electrophoresis

Sodium dodecyl sulfate-agarose gel electrophoresis (SDS-AGE) was performed on urine from all dogs, irrespective of UPC ratio, in order to determine diagnostic performance of SDS-AGE. All electrophoreses were performed with a semi-automated system (Hydrasys, Sebia Italia SRL, Italy). To satisfy linearity, urine supernatants which contained >2g/L of proteins were diluted to a 1:1 ratio with deionized water, as recommended by the manufacturer. Eighty μL of the final urine supernatant were mixed with 20μl of the additive provided by the manufacturer (Hydragel 5 proteinuria, Sebia). Five μL of the treated urine were loaded on the gels (agarose 50g/L), and the migration, staining with acid-violet and drying were conducted with the semi-automated system. The limit of sensitivity was 15 mg/L per band. Each gel contained 5 wells, the fifth one being filled with the manufacturer’s molecular markers (MM) solution (Molecular mass control, Sebia) to facilitate protein identification. This solution contained lysozyme (14.3 kDa), triose-phosphate isomerase (26.6 kDa), bovine albumin (66 kDa) and human IgG (150 kDa).

Gels were visually analyzed by the same operator (RL). Although they were not definitely identified and electromorphs may occur, those bands migrating at the same distance as bovine albumin and human IgG were considered to be albumin (albumin band, Ab) and immunoglobulin G (IgG band, IgGb) and recorded as such. The other bands were characterized as LMWb or HMWb according to their position relative to Ab.[14,29] An intensity score of 0 (no visible band), 1 (weak band) or 2 (intense band) was attributed to Ab to facilitate interpretation of the SDS-AGE pattern and comparison with the quantitative measurement of uAlb. The SDS-AGE pattern was considered normal if no band or only an Ab scored as 1 was present, because a weak Ab can be identified in healthy dogs [12,18,19,30]. Proteinuria was diagnosed of tubular origin if LMWb was detected on SDS-AGE and of glomerular origin if an Ab score of 2 or HMWb was present. When both LMWb and HMWb or an Ab score of 2 were observed, proteinuria was considered to be of mixed origin.

### Urinary markers

Urinary RBP, uAlb and uIgG concentrations were measured in 69 dogs, irrespective of UPC values. Determination of uNAG activity was performed in only 48 dogs as the urine storage length was considered inacceptable for the others [31].

Concentrations of the glomerular markers uAlb and uIgG were measured with commercially available canine-specific ELISA kits (Immunology Consultant Laboratory, Newberg, OR, USA). Tubular function was assessed by quantification of uRBP with a human ELISA (Immunology Consultant Laboratory) and uNAG activity by a colorimetric assay (Diazyme Laboratories, Poway, CA, USA) and spectrophotometer (Beckman DU-640 Spectrophotometer, Beckman-Coulter Inc., Belgium). All assays were performed according to the manufacturer’s instructions, and have been previously validated in dogs; the assay characteristics are reported elsewhere [16,32,33]. As spot urine specimens were used, concentrations of urinary markers were normalized to urine creatinine (c) concentration and expressed as a ratio in mg/g creatinine for uAlb/c and uIgG/c, in μg/g creatinine for uRPB/c and in U/g creatinine for uNAG/c.

In order to compare diagnostic performance of SDS-AGE with that of quantitative urinary markers, “health” decision thresholds for the latter were determined. Thus, reference intervals (RI) for the urinary marker ratios were determined in non-proteinuric DDB dogs. The upper limits of these RI were used as thresholds, and values above these limits were considered abnormal.

### Statistical analysis

Correlations between UPC, storage duration, SDS-AGE and quantitative urinary marker results were assessed with Spearman’s correlation coefficients and their corresponding significance. Potential differences between UPC subgroups for urinary marker results were assessed by a Kruskal-Wallis test. All calculations were performed using computer software (Systat 13, SPSS Inc., Chicago, IL, USA). For all analyses, p-value <0.05 was considered significant.

Urinary markers RI were determined according to international human and veterinary guidelines [34,35]. Possible outliers were detected by visual inspection of histograms and Tukey’s criterion and excluded from the calculation of reference limits. Normality of distribution of the native or transformed values was tested with the Anderson-Darling test. Reference limits were determined using the parametric robust method after logarithmic transformation. The 90% confidence intervals (CI) were calculated with a bootstrap method. Reference Value Advisor (V 2.1) was used to determine the RI and their CI [36]. Dogs with results below the detection limits of the assay were arbitrarily assigned the value of the detection limit for RI determination. Additionally, when the number of animals was < 20, only median and range of data are reported, as RI determination on small effective has been reported to be unreliable, and the maximal value was chosen as the decision threshold [37].

Correlations between the presence of LMWb and uNAG/c or uRBP/c, between the presence of IgGb and uIgG/c and between the intensity score of Ab and uAlb/c were analyzed with Spearman’s correlation coefficients. When correlations were significant, further analyses were performed to determine the diagnostic performance of these electrophoretic bands in identifying elevated urinary marker ratios. True positive (TP), false positive (FP), true negative (TN) and false negative (FN) were determined depending on the aforementioned “health” thresholds for urinary markers and the presence or scores of bands identified on SDS-AGE. Presence of LMWb was compared to uRPB/c and uNAG/c, presence of an IgGb was compared to uIgG/c and finally Ab scores were compared to uAlb/c. These results were used to determined the sensitivity, specificity, positive and negative likelihood ratio (LR+ and LR–, respectively) and their respective 95% CI with the Predictive Value Advisor freeware (http://www.biostat.envt.fr/ppvnpv/#) [38].

## Results

### SDS-AGE

The median urine storage time before SDS-AGE analysis was 12 months, with a minimum of 5 months and a maximum of 24 months. Storage duration was not correlated with any SDS-AGE finding. In 21 dogs the urine proteins concentrations were over 2g/L and needed to be diluted prior to SDS-AGE.

**Fig 4:**
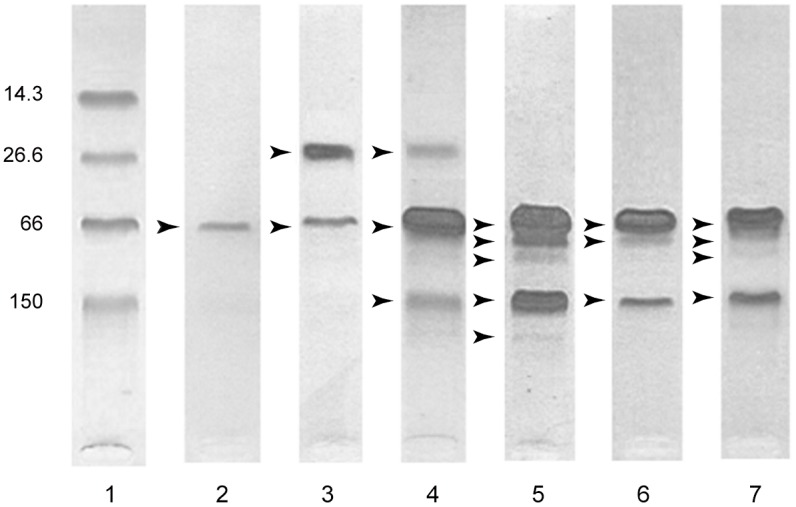
Example of the different results for SDS-AGE performed on urine. Lane 1 represents the migration of molecular markers, lane 2 a normal pattern, lane 3 a tubular pattern, lane 4 an abnormal mixed pattern, lane 5 an abnormal glomerular pattern from the non azotemic dog with confirmed familial GN. Lanes 6 and 7 both represent glomerular pattern from dogs with stage II and III CKD respectively. The various protein bands identified are indicated by arrowheads. The numbers on the left side correspond to molecular weight of molecular markers in kDa.

Considering the 100 clinically healthy dogs, the only LMWb observed was located at approximately 25 kDa (Fig 4); this band was obtained after analyzing urine from 25 entire male dogs. Most of these dogs had variable numbers of spermatozoids on sediment microscopic examination. The Ab score was 0, 1 and 2 in 16, 43 and 41 dogs, respectively. All specimens that needed to be diluted had a Ab score of 2. When all the results were taken into account all results, 4 different HMWb were identified and located at approximately 80, 100, 150 and 190 kDa, respectively (Fig 4). In 49 dogs, HMWb was detected. At least an IgGb was identified in the remaining dogs; and one, two or three additional HMWb were identified in 19, 17 and 10 dogs, respectively. Three distinct HMWb in addition to the IgGb were apparent int he female dog that underwent renal biopsy. The Final interpretation of the SDS-AGE results was absence of abnormal proteinuria in 39 dogs, and tubular, glomerular and mixed proteinuric pattern in 9, 36 and 16 dogs, respectively.

No LMWb was identified in the two dogs suffering from proteinuric CKD. Both had an Ab score of 2 and a clearly defined IgGb. In Additional to a heavy Ab, the dog with stage II CKD had 2 distinct HMWb, while the stage III CKD dog displayed 3 HMWb. A glomerular pattern of proteinuria was attributed to both dogs (Fig 4).

Detailed information regarding the distribution of SDS-AGE proteinuria patterns, according to the IRIS staging system for UPC, is reported in Table 1 [28]. In 34 out of 39 dogs with normal urine SDS-AGE pattern, UPC was≤0.2. No dog with a tubular proteinuria pattern had a UPC>0.5, whereas 34 out of 54 dogs with glomerular or mixed proteinuria patterns had a UPC>0.5. Number of HMWb and intensity of Ab showed strong positive correlation with UPC (p<0.0005). Presence of the 25kDa LMWb was not correlated with UPC.

**Table 1:** Correspondence between final SDS-AGE interpretation and UPC in 102 DDB dogs.

SDS-AGE Interpretation	UPC		
	≤0.2	>0.2 and ≤0.5	>0.5
Normal pattern	34	4	1
Tubular pattern	7	2	0
Glomerular pattern	6	11	21
Mixed pattern	0	3	13

UPC, urine protein-to-creatinine ratio; Final SDS-AGE: final interpretation of the urine protein electrophoretogram.

### Quantitative urinary markers

Median duration of urine storage was 3 months, with a minimum of 2 months and a maximum of 15 months for 5 specimens, and was not correlated with the urinary marker ratios (Table 2). Urinary RBP/c, uAlb/c and uIgG/c were measured in 69 dogs (67 healthy and 2 CKD dogs) while uNAG/c activity was determined in 48 animals (47 healthy and 1 CKD dogs). Median values and [range] for uNAG/c, uRPB/c, uAlb/c and uIgG/c were: 7.00 U/g, [2.22–41.19]; 36.20 μg/g, [<14.11–1730.00]; 113.00 mg/g, [2.00–2836.00] and 12.28mg/g [0.26–421.98], respectively.

**Table 2:** Correlations (r) between urinary markers, UPC and storage duration.

Variable	UPC	Storage	uRBP/c	uAlb/c	uIgG/c
uNAG/c (n = 48)	0.407*	-0.249	0.393*	0.229	0.364*
uRBP/c (n = 57)	0.748**	0.052	-	0.740**	0.618**
uAlb/c (n = 69)	0.958**	0.238	0.229	-	0.821**
uIgG/c (n = 69)	0.847**	0.098	0.618**	0.821**	-

UPC, urine protein-to-creatinine ratio; Storage, storage duration; uALB/c, urinary albumin-to-creatinine ratio; uRBP/c, urinary retinol binding protein-to-creatinine ratio; uNAG/c, urinary N-acetyl-β-D-glucosaminidase-to-creatinine ratio.

* Significant correlation p<0.05

** Significant correlation p<0.0005

Considering the 100 healthy DDB dogs, UPC was ≤0.2, > 0.2 and ≤ 0.5, and >0.5 in 30, 11 and 26 dogs for which uRBP/c, uAlb/c and uIgG/c were determined, and in 17, 11, and 19 dogs for which uNAG/c was available, respectively. Twelve dogs (all with UPC≤0.2) had an uRBP below the detection limit. The upper and lower reference limits and 90%CI of the RIs determined in non-proteinuric dogs are given in Table 3. Because of the small number of non-proteinuric dogs available for determination of uNAG/c, only median and range are reported in this table. For the clinically healthy dog that underwent kidney biopsy, uRBP/c, uAlb/c and uIgG/c were determined and only uAlb/c (1758 mg/g) and uIgG/c (48.46mg/g) were abnormal.

**Table 3:** Medians and ranges for uNAG/c, uRPB/C, uAlb/g and uIgG/c, and lower and upper limits of the reference intervals for uRPB/C, uAlb/g and uIgG/c determined in non-proteinuric DDB dogs.

Analyte	Dogs (n)	Median (Range)	2.5^th^ Percentile (90% CI)	97.5^th^ Percentile (90% CI)	Normality (p)
**uNAG/c** (U/g)	17	5.74 (2.75–13.396)	ND	ND	0.003
**uRBP/c** (μg/g)	30	21.74 (BDL-76.56)	<2.77 (ND)	82.74 (51.86–132.24)	<0.001
**uAlb/c** (mg/g)	30	19.30 (2.06–96.65)	1.82 (1.04–3.27)	233.72 (143.58–476.58)	0.001
**uIgG/c** (mg/g)^a^	28	1.41 (0.26–23.60)	0.15 (0.09–0.3)	9.78 (4.82–20.80)	<0.001

BDL, below detection limit; ND, not determinable

^a^: Excluded outliers = 54.99 and 68.33 mg/g

In the dog with stage III CKD, all the urinary marker results were elevated (uNAG/c = 15.05 U/g, uRBP/c = 1021 μg/g, uALb/c = 1437mg/g, uIgG/c = 89 mg/g), while in the stage II CKD dog, only uAlb/c (946 mg/g) and uIgG/c (71 mg/g) were elevated.

**Fig 5:**
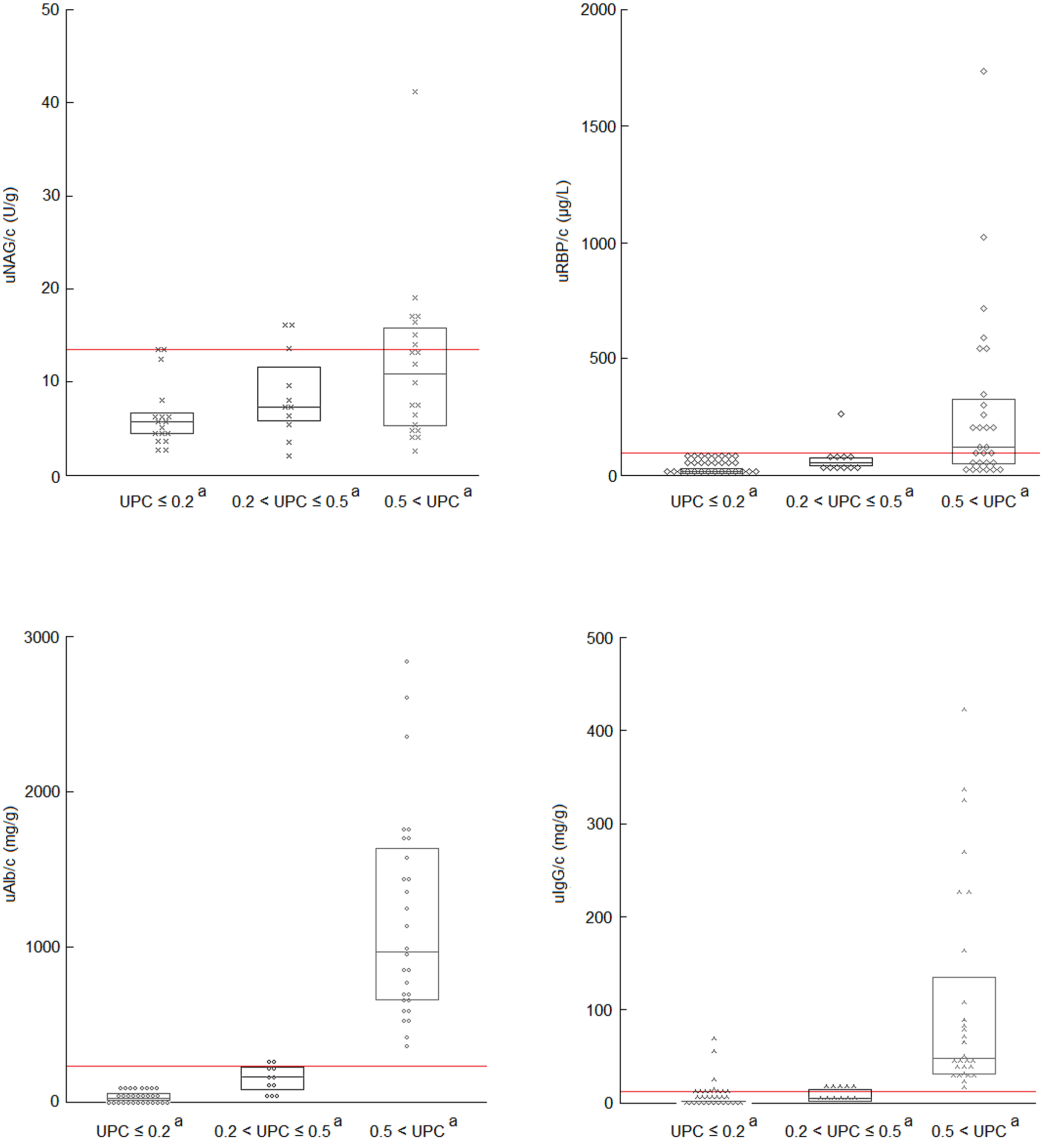
Distribution of the values for the four urinary marker ratios depending on UPC subclass. The bottom and top of the box indicate the 25th and 75th percentile respectively and the horizontal line inside the box shows the median distribution. Red lines represent upper “health” thresholds. When the letter in superscript is similar, a significant difference exist between group (p<0.001)

The distributions of urinary markers ratios according to UPC subclasses and significant differences between groups are shown in Fig 5. All ratios were positively correlated to UPC (Table 2). All ratios were below the “health” thresholds in 26 out of 30 non-proteinuric dogs and in 4/11 borderline proteinuric dogs. Only one borderline proteinuric dog had both elevated uAlb/c and uIgG/c. All proteinuric dogs had abnormally elevated uAlb/c and uIgG/c when compared to the upper limits of their respective RIs, but in only 6 out of 28 were all 4 markers above their respective thresholds (Fig 5).

### Diagnostic performance of SDS-AGE

Strong correlations were demonstrated between uAlb/c and Ab score (r(67) = 0.898, p<0.0005) and between uIgG/c and IgGb (r(67) = 0.732, p<0.0005). Presence of the LMWb was not correlated with uRBP/c nor uNAG/c.

With a threshold of 234mg/g for uAlb/c, 29 out of 31 dogs with increased uAlb/c also had an Ab score of 2, while 35 out of 38 dogs with uAlb below the threshold had a Ab score of 0 or 1. Therefore, the sensitivity, specificity, LR+ and LR- (95% CI) of SDS-AGE to correctly identify abnormal uAlb/c was 94% (85–100%), 92% (83–100%), 11.85 (3.90–36.03) and 0.07 (0.02–0.28), respectively.

With a threshold of 10 mg/g for uIgG/c, 29 of the 36 dogs with elevated uIgG/c also had a distinct IgGb, while 25 of the 33 dogs with normal uIgG/c showed no IgGb on SDS-AGE. The sensitivity, specificity, LR+ and LR- (95% CI), of SDS-AGE to predict dogs with abnormal uIgG/c was 90% (80–100%), 74% (60–88), 3.43 (1.97–5.98) and 0.13 (0.04–0.40), respectively.

## Discussion

In the present study, an intense Ab and/or HMWb, as well as elevated uAlb/c and uIgG/c were observed in the urine of a large proportion of clinically healthy DDB dogs and in the two proteinuric CKD dogs. These findings were highly correlated with UPC. Close correlations were also found between some SDS-AGE findings and quantitative urinary markers and diagnostic performance of SDS-AGE to predict dogs with abnormal uAlb/c and uIgG/c were good. Furthermore, renal biopsies obtained from two dogs revealed similar lesions compatible with histological features described in canine familial GN.

As the analyses were performed on urine specimens stored at -80°C, a variable storage length could have led to pre-analytical variations and biased results. Also, the urine specimens were kept at -20°C for less than 24h during their shipment from the site of collection to the Laboratoire Central de Biologie Médicale of the National Veterinary School of Toulouse. Nevertheless, all pre-analytical and analytical conditions were standardized as much as possible. Collection, processing and storage temperature were indeed similar in all dogs [2,27]. Very little information is available regarding the stability of urinary proteins evaluated by SDS-AGE. It was recently demonstrated that appearance or increased intensity of the IgGb was visible on SDS-AGE profiles from proteinuric and non-proteinuric dogs after storage for 15 days at 20°C, and that the SDS-AGE profiles were not affected by a storage at -80°C for up to 6 months [unpublished data]. Additionally, human studies showed that polyacrylamide gel-electrophoresis patterns of urine proteins were not affected by long periods of storage at -70°C [39,40]. It is therefore unlikely that the electrophoretic patterns were markedly altered by storage period, although the number of dog with an IgGb might have been overestimated because of the initial storage at -20°C. Most quantitative urinary markers have been demonstrated to be stable for several months in canine specimens stored at -80°C, except for uNAG, for which controversies exist. One study indeed showed that uNAG/c decreased significantly after 12 months of storage at -80°C, while, in another study, no difference was seen after 1 year of storage at -20°C or -80°C [16,31]. To avoid erroneous results, no urine specimen stored for more than 4 months at -80°C was used to determine uNAG activity in the present study. Additionally, no significant correlation was found between storage duration and the values of urinary markers.

Early identification of glomerular abnormalities is of major importance in breeds predisposed to familial glomerulopathy, in order to improve breeding policy. The gold standard for evaluating the nature and severity of canine glomerular lesions is histopathological examination of kidney biopsies [10,11,41]. Nevertheless, this is an invasive and costly procedure, especially as a screening test. Thus, non-invasive urinalyses are attractive options to screen dogs at risk. Even if information regarding values for urinary marker ratios in healthy dogs is available in the context of comparative studies [16,17,25,33], no RI have been established that could be used as “health” threshold values to screen dogs at risk for glomerular disease. We therefore decided to determine breed specific RI for urinary marker ratios, by including non-proteinuric DDB dogs. We cannot exclude that in some of the borderline and proteinuric dogs renal function may have been completely normal, or that some dogs with normal UPC may have suffered from early renal lesions. However, as most healthy dogs have a UPC≤0.2 [42–45], it is likely that renal function in most of the non-proteinuric DDB dogs was normal. Although the number of non-proteinuric dogs was small and might have produced questionable thresholds [37], the values used in the present study were nevertheless comparable to those previously published for healthy dogs [16,17,25,33]. Furthermore, the urinary markers were strongly correlated with UPC, as has been shown in most canine studies [16,17,25,26,33,46]. It is thus likely that the upper limits of the RIs determined in the non-proteinuric subgroup are close to the “health” thresholds for the breed.

In the present study, glomerular or mixed SDS-AGE patterns as well as increased uAlb/c and uIgG/c were found in all healthy DDB dogs with proteinuria and in some dogs with borderline proteinuria. It is thus very likely that most of these dogs suffered from underlying glomerular lesions. Indeed, both glomerular patterns on urine protein electrophoresis and elevated uAlb/c and uIgG/c have been shown to be correlated with presence of glomerular lesions in the dog [14,16,20,25,47]. Additionally, all these SDS-AGE profiles were similar and alike those obtained in the 2 dogs diagnosed with a primary familial glomerular disease based on renal biopsies.

Despite the significant difference in uNAG/c and uRBP/c between UPC subgroups, only some dogs with results indicative of glomerular dysfunction also had concomitant uNAG/c (35%) or uRBP/c (56%) above the “health” thresholds. Interestingly, these dogs were amongst those with the highest UPC and proteinuria has been demonstrated to gradually impair proximal tubular epithelial structure and function [48–50].

A higher proportion of proteinuric DDB dogs had increased uRBP/c rather than increased uNAG/c. This might reflect the fact that mild to moderate proteinuria mainly competes with RBP reabsorption [21,23,49], rather than induces tubular lesions [22,49].

In the present study, only one LMWb of approximately 25 KDa was identified on SDS-AGE and visualization of this band was neither correlated with UPC, uNAG/c nor uRBP/c. However, this finding is not surprising given the fact that the maximal concentration of uRBP measured in the included dogs (*i*.*e*. 4.87 mg/L of urine) was well below the analytical limit of detection of acid-violet staining. The diagnostic sensitivity of SDS-AGE could likely be improved if silver staining, which has a lower analytical reference limit (*i*.*e*. approximately 1 mg/L per band), was used [51]. Additionally, the only visible LMWb was exclusively observed in entire male dogs, and spermatozoids were detected on direct microscopic urinalysis in most of these dogs. Given the absence of correlation between this band and quantitative urinary markers, it seems unlikely that this band is of renal tubular origin. It might however correspond to urine contamination by seminal proteins. Indeed retro-ejaculation is known to occur frequently in dogs, and a similar LMWb has previously been visualized in urine from intact male dogs by SDS-AGE and characterized as a major prostatic protein: arginine esterase [52,53].

Although visualization of a LMWb could not be correlated towith quantitative markers of tubular function, the semi-quantitative scoring adopted to interpret electrophoretic Ab and the presence of an IgGb were however correlated with uAlb/c and uIgG/c. Based on diagnostic performances of SDS-AGE obtained in the present study, the observation of a normal SDS-AGE pattern in a DDB dog is strongly suggestive of normal uAlb/c and uIgG/c, and thus of a normal glomerular permselectivity [23]. These results are in accordance with two previous studies in dogs showing that the sensitivity of urine protein gel-electrophoresis to detect glomerular lesions was very good (97 to 100%) [14,20], and are therefore especially useful for DDB dogs with normal or borderline UPC according to IRIS staging system [28]. While dogs with persistent UPC > 0.5 are considered to have a pathological proteinuria, SDS-AGE might help in screening apparently healthy DDB dogs with borderline proteinuria. The specificity of SDS-AGE to predict glomerular lesions was reported to be much lower (40 to 60%) than the values found here to identify elevated uAlb/c and uIgG/c. This difference may partly be attributed to the semi-quantitative scoring used for albumin: indeed, whereas the presence of an isolated weak Ab was considered normal in the present study, it was considered secondary to glomerular lesions in other studies [14,20]. Another explanation for these discrepancies could be the temperature used to store urine specimens in these studies, as it seems that IgGb may appear on a urinary SDS-AGE pattern if urine was stored at -20°C [unpublished data]. This phenomenon could also explain the lower specificity of SDS-AGE to predict elevated uIgG/c than elevated uAlb/c that was found in the present study.

## Conclusion

In conclusion, the results of the present study strongly support the hypothesis that otherwise healthy proteinuric DDB dogs have underlying glomerular lesions, which are associated with tubular dysfunction only in dogs with the highest UPC. The diagnostic performance of SDS-AGE to predict DDB dogs with elevated uAlb/C and uIgG/C was found to be good. This method could therefore be a reliable screening option to rule out glomerular lesions in DDB dogs for which UPC is repeatedly within the borderline range. Nevertheless, longitudinal studies of the quantitative and qualitative characteristics of proteinuria, combined with renal histopathological description, are required to complete the present results, in order to assess the true prevalence of glomerular lesions within the breed and its relationship with the familial glomerulonephropathy and find out if DDB could be a spontaneous model of glomerular disease.
